# Editorial: Collaborative Efforts for Understanding the Human Brain

**DOI:** 10.3389/fninf.2019.00038

**Published:** 2019-05-29

**Authors:** Sook-Lei Liew, Lianne Schmaal, Neda Jahanshad

**Affiliations:** ^1^Chan Division of Occupational Science and Occupational Therapy, University of Southern California, Los Angeles, CA, United States; ^2^Keck School of Medicine, Mark and Mary Stevens Neuroimaging and Informatics Institute, University of Southern California, Marina del Rey, CA, United States; ^3^Orygen, The National Centre of Excellence in Youth Mental Health, Parkville, VIC, Australia; ^4^Centre for Youth Mental Health, The University of Melbourne, Melbourne, VIC, Australia

**Keywords:** neuroinformatics, neuroscience, neuroimaging, genetics, omics and big data analysis, collaborative science

Advancements in the technologies and methods used to study the brain have improved our capability to collect, share, and analyze large, detailed, datasets including brain imaging, genetic, andv behavioral data. This data-rich environment has allowed researchers to study the complex relationships between the structure and function of the brain throughout the lifespan with different behaviors or even clinical disease states. Today, research aimed at understanding the human brain necessitates new collaborative efforts that bring together domain experts across neuroscience, computer science, biology, engineering, statistics, medicine, and clinical practice in order to maximize the impact of these large and diverse datasets.

In this Research Topic, we highlight many novel and exciting aspects of this collaborative effort to study the human brain. This issue begins by providing perspectives on the evolving field of reproducible science, which is a main goal for collaborations in the field, and also highlights the emerging use of web-based applications for bringing together researchers from around the world. Next, several papers describe new large-scale neuroinformatics platforms that centralize data collection, simplify data management, implement rigorous quality control, and integrate complex multi-modal neuroimaging, genetic, and behavioral datasets. These are followed by several papers describing new software analysis pipelines for neuroimaging data (e.g., PET imaging and stroke MRI imaging), which provide flexible yet standardized and reproducible tools for data analysis. This issue also contains several reports comparing different ways of collecting and analyzing large, multi-site data, and provides new insights into best practices for multi-site diffusion MRI acquisition, neuroimaging data analysis, and imaging genetics analyses. Finally, several papers introduce new methods for analyzing multi-site data, including decentralized voxel-based analyses, hybrid mesio-temporal lobe segmentation, and analyses for post-traumatic epilepsy data. Overall, these papers demonstrate the breadth of work focused on bringing researchers together to decode the mysteries of the human brain.

## Perspectives on Reproducible Science

As research becomes more collaborative, excitement around issues of reproducible and open science are growing. In this special issue, several authors provided insight on how to perform reproducible and collaborative research using cutting-edge open-source tools. For example, Kennedy et al. describe work emerging from ReproNim: A Center for Reproducible NeuroImaging Computation and detail principles of reproducible science, focusing on publications that can be re-executed. In doing so, they highlight a number of ReproNim tools for data management, analysis, and reporting. Building on this, Keshavan and Poline share their perspective on a new wave of collaborative science happening over web-based platforms. They outline how the internet allows for improved sharing of data and research projects, and they share numerous web-based tools for everything from crowd-sourced data analysis to collaborative writing.

## Frameworks and Platforms to Facilitate Data Sharing

The implementation of these principles for open science is also detailed in several papers describing new neuroinformatic platforms for managing and harmonizing large multi-modal datasets. First, Mohaddes et al. described a neuroinformatics platform developed for the Canadian Consortium on Neurodegeneration in Aging (CCNA). This framework uses the LORIS data management system and supports the acquisition, storage, curation and dissemination of imaging, genetic, clinical, and biospecimen data related to aging. Next, Das et al. describe an integrated “-omics” framework, also using the LORIS platform, for analyzing multiple types of “-omics” datasets, including genomics, imaging, and behavior. Similarly, Vaccarino et al. describe the Ontario Brain Institute's Brain-CODE platform, which facilitates clinical, neuroimaging, and molecular data management, analysis and sharing in one consolidated, open-source platform. Finally, Rotenberg et al. describe a use case of the Brain-CODE platform from the Center for Addiction and Mental Health (CAMH). This platform provides an environment for centralized data capture, visualization, and analysis for psychiatric data. Importantly, all of these platforms and examples emphasize key issues in data management, such as privacy, data permissions, and quality control. They also all focus on integrating complex multimodal data sources in a manner that is easy to curate and share.

## Software Pipelines for Harmonized Analyses

While the neuroinformatics platforms aim to simplify data management, there are also attempts to develop software pipelines that will standardize data analysis. Funck et al. describe the APPIAN (Automated Pipeline for PET Image Analysis) toolbox, which allows for the robust, reproducible analysis of PET imaging data with many options for flexible processing. For stroke MRI data, Ito et al. describe the PALS (Pipeline for Analyzing Lesions After Stroke) toolbox, which supports large-scale lesion analysis and quality control with many user-defined options for analysis. Both are good examples of software pipelines that can harmonize data analysis across research sites and improve reproducibility of results.

## Harmonization of Protocols and Methods

In addition to new platforms and software, another important issue in collaborative research is to evaluate and compare different methods of data acquisition and analysis. To this end, a number of papers examined different methods for acquiring and analyzing data in order to harmonize data collection and analysis across research sites. Zavaliangos-Petropulu et al. compared six different diffusion tensor imaging (DTI) scanner acquisition protocols acquired across 47 different research sites from the Alzheimer's Disease Neuroimaging Initiative (ADNI). Although they found differences in diffusion metrics based on the imaging protocol, they were able to successfully pool the data across the sites and protocols into one cohesive dataset. Boedhoe et al. from the ENIGMA Obsessive-Compulsive Disorder Working Group used multi-site data from 38 research sites to compare statistical approaches to pooling MRI-derived measures, and found that for this type of data, mega-analytic approaches are favorable to meta-analytic analyses. Kochunov et al. compared different methods for estimating heritability from imaging genetics data using a host of tools across multiple datasets. They found that although the different methods yielded different results depending on the dataset and the approach, incorporating several homogenization steps prior to estimating heritability was effective in producing converging results across methods. Each of these important contributions not only shows how multi-site data can be affected by different methods, but also provides recommendations for improving harmonization of the data.

## Methods for Multi-Site Data Analyses

Finally, several papers described new methods for analyzing multi-site data. Gazula et al. proposed new decentralized methods for structural (voxel-based morphometry) and functional (dynamic functional connectivity) analyses, and compared this with standard centralized methods. They found that the decentralized methods worked equally well as centralized methods but are more flexible for use with multi-site data, opening the doors for large-scale collaborative analyses without bulky data-transfers. Kim et al. discussed a new hybrid template approach for automated segmentation of mesiotemporal structures, including the hippocampus, amygdala, and parahippocampal gyrus, which reliably performs better than existing segmentation methods across multiple datasets. Finally, Duncan et al. describe preliminary methods and results for analyzing multi-site data in individuals with epilepsy following traumatic brain injury from the multi-site Epilepsy Bioinformatics Study for Antiepileptogenic Therapy (EpiBioS4Rx) study, which collects MRI, EEG, and intracranial EEG from humans and animals. Overall, these papers reveal new methods specific for multi-site data analysis.

## Conclusions and Future Directions

Work presented in this Research Topic collectively highlights the growing trend for collaborative efforts for the neurosciences. These collaborations come in the form of developing tools for external researchers to access or contribute data, developing methods that are confirmed to be robust across MRI datasets and acquisitions, and empirically testing harmonization methods for diverse datasets. Through the tools, methods, and results presented in this issue and beyond, researchers around the world are teaming up to ensure this new era of science provides robust, reliable and internationally meaningful findings to drive the understanding of the human brain forward.

## Author Contributions

All three authors served as editors of this Research Topic and contributed to the conceptualization of this Research Topic. The editorial was drafted by SL-L and revised and reviewed by LS and NJ.

### Conflict of Interest Statement

The authors declare that the research was conducted in the absence of any commercial or financial relationships that could be construed as a potential conflict of interest.

